# Comparative Study of Dentinogenesis Imperfecta in Different Families of the Same Topographical Region

**DOI:** 10.5005/jp-journals-10005-1015

**Published:** 2009-12-26

**Authors:** MK Jindal, Sandhya Maheshwari, Radhika Verma, Mohd Toseef Khan

**Affiliations:** 1 Chairman and Associate Professor, Department of Pedodontics, Faculty of Medicine, Aligarh Muslim University Aligarh-202002, Uttar Pradesh, India; 2 Professor, Department of Orthodontics, Faculty of Medicine, Aligarh Muslim University, Aligarh-202002, Uttar Pradesh, India; 3 Tutor, Department of Pedodontics, Faculty of Medicine, Aligarh Muslim University, Aligarh-202002, Uttar Pradesh, India; 4 Junior Resident 1st Year (MDS), Department of Orthodontics, Faculty of Medicine, Aligarh Muslim University, Aligarh-202002 Uttar Pradesh, India

**Keywords:** Dentin hypoplasia, shell teeth, dentinogenesis imperfecta, autosomal dominant, mesodermal defect.

## Abstract

Dental hard tissue is subject to variety of disorders. Dentinogenesis Imperfecta is one such disorder attributed to heredity. It is known to be an autosomal dominant trait. Teeth with such ‘imperfect’ dentin are liable to be weak and discolored. The disease has variable penetration and therefore can be expressed as a range of phenotypic manifestations from mild discoloration and chipping to frank attrition and multiple pulp canal exposures. Here we present a comparative study of a series of cases from different families of one topographical region with widely different presentation and histories that are characteristic of this disease.

## INTRODUCTION

Dentin, which is a bone-like substance that makes up the protective middle layer of each tooth, is formed by cells, the odontoblasts. These differentiate from ectomesenchymal cells of the dental papilla following an organizing influence that emanates from cells of the internal dental epithelium. The odontoblasts produce an organic matrix that later becomes mineralized to form dentin.^1^ For the formation of dentin, the DSPP gene provides instructions for making three proteins that are essential for normal tooth development. DSPP mutations alter the proteins made from the gene, leading to the production of abnormally soft dentin. Teeth with defective dentin are discolored, weak and more likely to decay and break.^2^

Dental hard tissue is subject to variety of disorders. Dentinogenesis Imperfecta is one such disorder attributed to heredity. It is known to be an autosomal dominant trait with variable expressivity,^3^ due to either allelic mutations in the DSPP gene or as part of a bigger problem involving osteogenesis imperfecta or even hearing loss.^2^-^4^

Dentinogenesis imperfecta (DI) was probably first recognized by Barret in 1882.^5^ The term was coined by Robert and Schor in 1939.^4^ Witkop reported that it was most common autosomal dominant disease affecting westerners.^6^,^7^

**Fig. 1.**
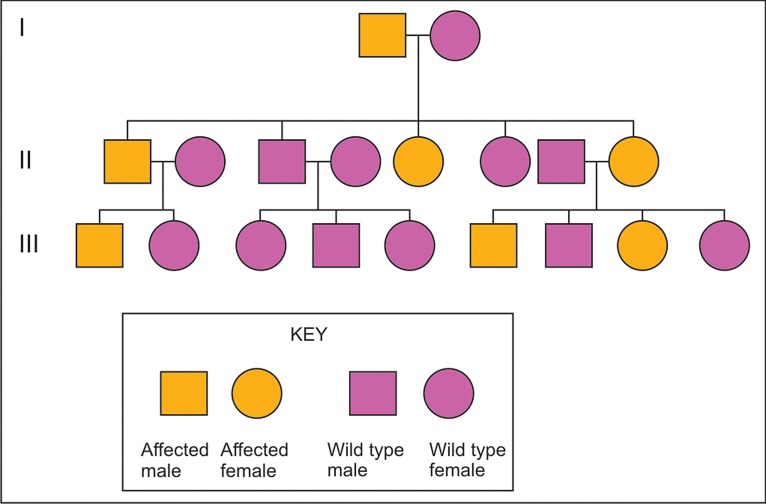
Autosomal dominant pattern of inheritance

Dentinogenesis imperfecta is reported to have an incidence of 1 in 6,000 to 1 in 8,000. As DGI and DD are inherited in an autosomal dominant fashion, there is a 50% chance that a child born to an affected parent will themselves be affected (Fig. 1).^2^

The affliction can phenotypically be manifested as just a mild discoloration with some attrition whether localized and affecting a few teeth or generalized and affecting multiple teeth. It has been known to affect the dentin of both primary and permanent dentitions, sometimes one more than the other. The affliction may span generations or more than one members of the same generation or even just one individual. Because of the clinical discoloration of teeth, this condition has also been named as hereditary opalescent dentin.^4^

Dentinogenesis imperfecta has been divided into two broad types. Type I, where the dentin abnormality occurs in patients with concurrent osteogenesis imperfecta (OI), primary teeth are more severely affected than permanent teeth. In type II patients have only dentin abnormalities and no bone disease. This type may or may not be associated with hearing loss. Also included in type II according to the new classification is the erstwhile type III or the Brandywine type (Discovered in a Triracial population in Brandywine, Maryland) where like the classical type II, only dental defects (and not OI) occur, and dentin dysplasia type II.

Features of the Brandywine type that are not seen in classical type I and type II include multiple pulp exposures, periapical radiolucencies and a variable radiographic appearance.

Clinically, all types share numerous features.^4^ In both dentitions the teeth exhibit an unusual, translucent opalescent appearance with color variation from dirty white, yellow brown to blue-gray or gray. The entire crown appears discolored because of the abnormal underlying dentin. Although enamel is structurally and chemically normal, it fractures easily resulting in rapid wear. This is due to the absence of microscopic scalloping present at DEJ. Overall the tooth morphology is unusual for its excessive constriction at the cementoenamel junction, giving crown a Tulip or bell shaped appearance.

Radiographically type I and II exhibit identical changes.^4^-^6^ Opacification of dental pulp occur due continued deposition of abnormal dentin. The short roots and bell shaped crowns are also obvious on radiographs. In type II (Brandywine type/erstwhile type III) the dentin appears thin and pulp chambers and root canals are extremely large giving appearance of thin dentin shells-hence the name shell teeth.

Microscopically the dentin contains fewer, but larger and irregular, dentinal tubules. Pulpal space is nearly completely replaced over time. Enamel appears normal but DEJ is smooth instead of scalloped, hence the easy chipping.

Treatment is directed toward protecting tooth tissue from wear and toward improving the aesthetic appearance of teeth. Generally fitting with full crowns at an early age is the treatment of choice. These teeth should not be used as abutments because the roots are prone to fracture.^4^

Here we have tried to present a comparative study of a series of 7 cases from different families of one topographical region (plains of Western Uttar Pradesh, India) with widely different histories and presentation that are characteristic of this disease.

## CASE 1

**Fig. 2.**
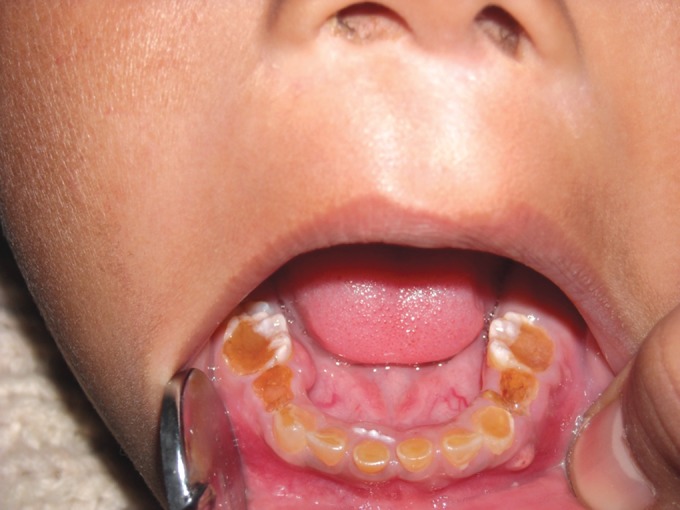
Mandibular teeth showing attrition in 4 years old female (case 1)

**Fig. 3.**
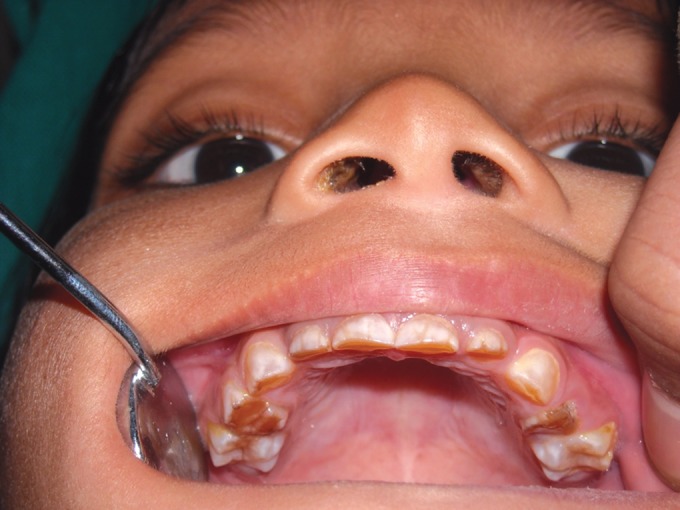
Maxillary teeth showing attrition in 4 years old female (case 1)

A 4 years old female patient reported to the Department of Pedodontics, Dr Ziauddin Ahmad Dental College, and Hospital, Aligarh Muslim University, Aligarh, Uttar Pradesh, India, with complains of discolored teeth and pain in lower jaw and wearing away of all teeth (Figs 2 and 3). She was accompanied by her father.

Her father gave history of the discoloration since the teeth erupted into oral cavity.

**Fig. 4.**
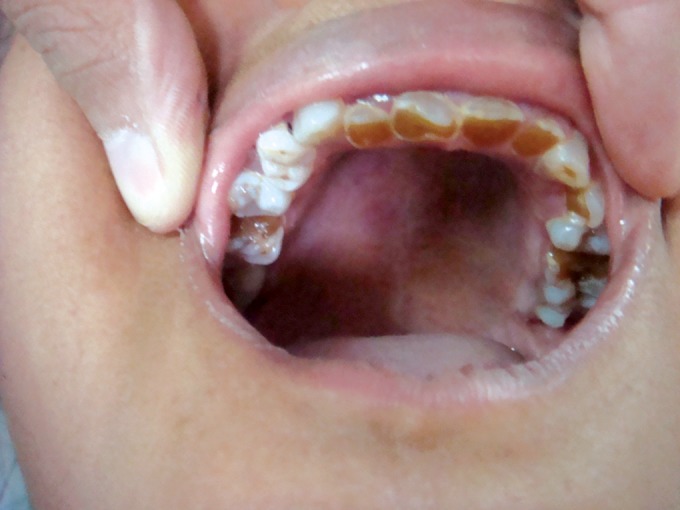
Maxillary teeth showing attrition in the maternal aunt of the child (case 1)

**Fig. 5.**
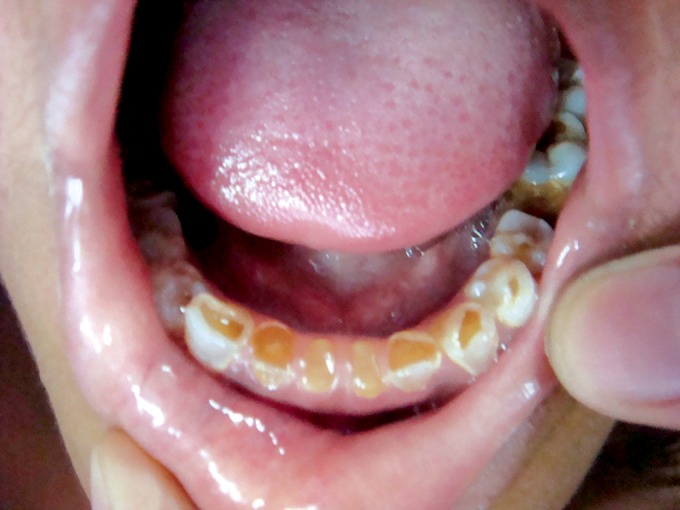
Mandibular teeth showing attrition in the maternal aunt of the child (case 1)

The family history revealed that her mother too had the same problem. On further investigation it was revealed that the condition also afflicted her maternal grandfather, two aunts (Figs 4 and 5) and their sons. The mother and her sisters due to lack of awareness, had never visited any dentist for proper treatment which led to gradual loss of tooth substance. No history of bone abnormality was revealed, in any of the family members.

Clinical examination revealed severe loss of enamel and dentin. Teeth revealed hard brown dentin with worn incisal and occlusal surfaces, in some teeth up to the gingival level. Mandibular first molars were exposed and there was abscess in relation to mandibular right first molar.

### Radiographic Examination

**Fig. 6.**
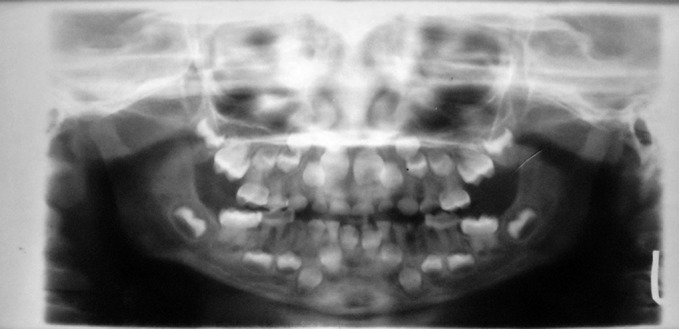
Orthopentamogram of the child (case 1), showing obliterated pulp chambers in deciduous and permanent teeth

**Fig. 7.**
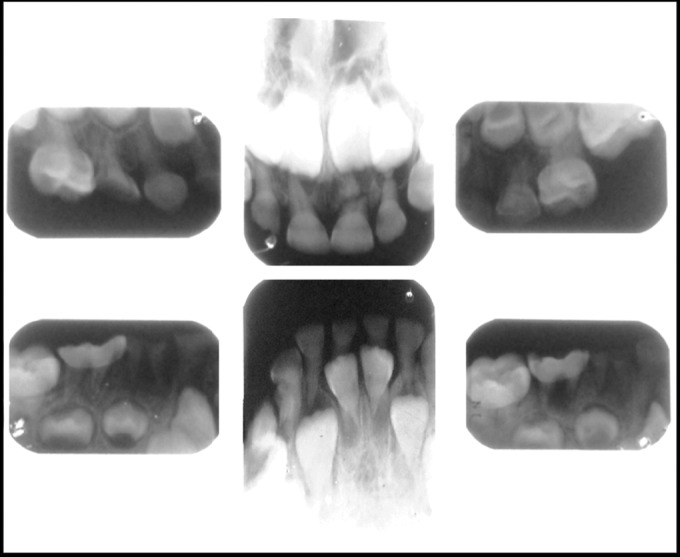
IOPA radiographs of the child (case 1) showing obliterated pulp canals of deciduous and permanent teeth

Orthopantomograph (Fig. 6) revealed obliterated pulp chambers in most of teeth and constricted in some teeth. Then we went for full mouth IOPA radiographic survey which also showed the same findings (Fig. 7).

## CASES 2 AND 3

**Fig. 8.**
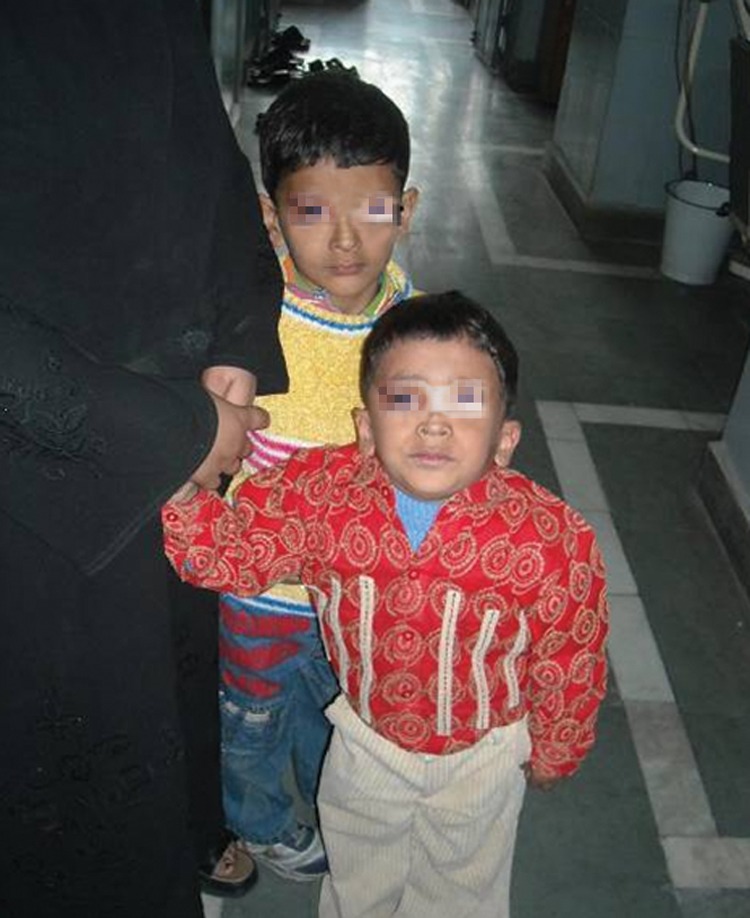
Brothers aged 5 and 6 (case 2 and 3 respectively)

Two brothers, aged 6 and 7 years (Fig. 8) came to our clinic accompanied with their mother with the complaint of all the teeth being rotten. As per history obtained from the mother the teeth started getting discolored and rotting soon after teething, in both her sons cases, and the new permanent teeth were following the same pattern. She admitted that as a family they were sometimes negligent of dental hygiene but none other in their family, from either hers’ or her husbands’ side, suffered from such a bad condition of teeth.

**Fig. 9.**
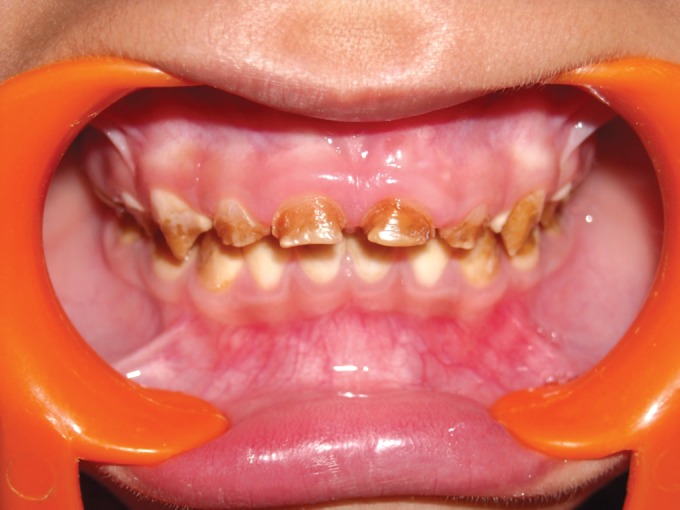
Photograph of 6 years old male (case 2) showing attrition in the maxillary deciduous

**Fig. 10.**
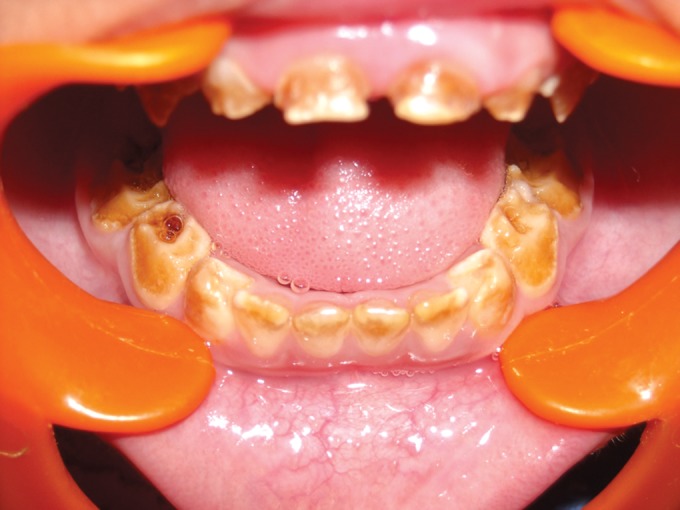
Photograph of the 6 years old male (case 2) showing attrition of both the deciduous and permanent teeth of dentition the mandibular dentition

Clinically, there was extensive and generalized involvement of all the teeth in both the cases with both deciduous and permanent dentitions seen to be involved. The elder brother's teeth (Figs 12 and 13) was more severely affected than the younger (Figs 9 and 10) and multiple pulp exposures of the anterior deciduous teeth observed. The enamel had started chipping off from even the newly erupted permanent teeth.

There was no pain or swelling associated with any tooth.

### Radiographic Examination

**Fig. 11.**
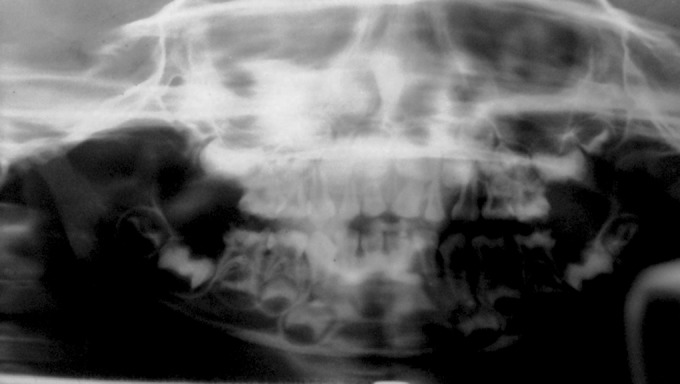
Orthopentamogram of the child (case 2) showing large pulp chambers and ‘shell like teeth’

Orthopantomograph revealed similar finding for both the cases [Figs 11(case 2) and 14 (case 3)]. Enlarged pulp chambers in all teeth both permanent and deciduous were seen.

## CASE 4

This patient, a 7 years old female, reported to the Department of Pedodontics complaining of pain in the upper right back tooth, dirty yellow teeth and wearing away of all teeth (Figs 15 to 17). She was accompanied by her father.

**Fig. 12.**
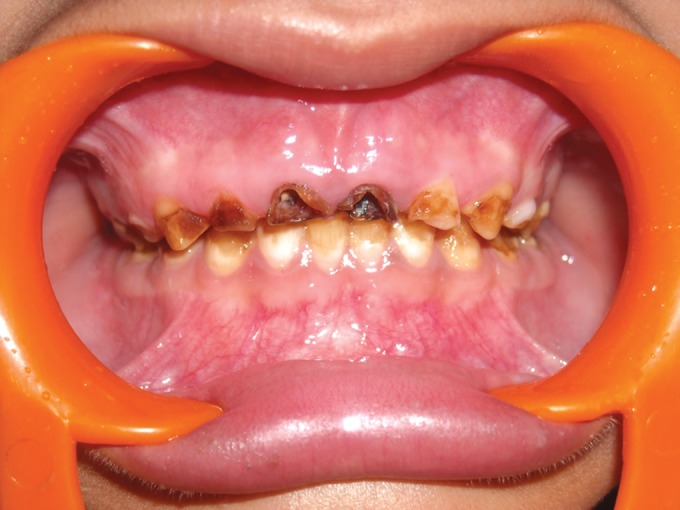
Photograph of 7 years old male (case 3) showing gross attrition and open canals in the maxillary deciduous dentition

**Fig. 13.**
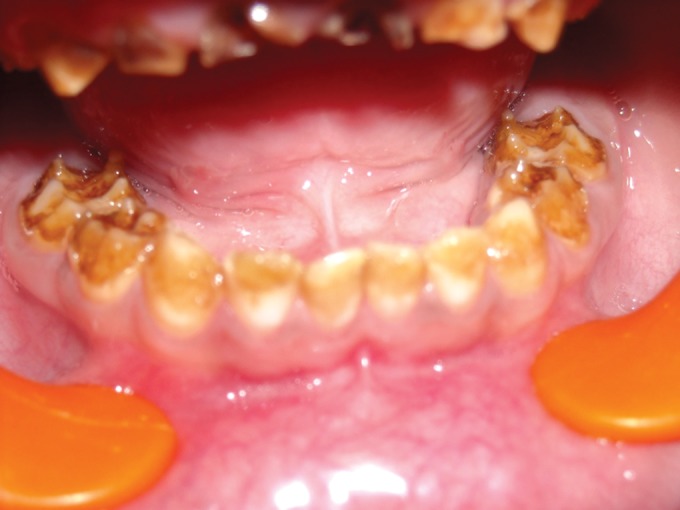
Photograph of the 7 years old male (case 3) showing attrition of both the deciduous and permanent teeth of the mandibular dentition

**Fig. 14.**
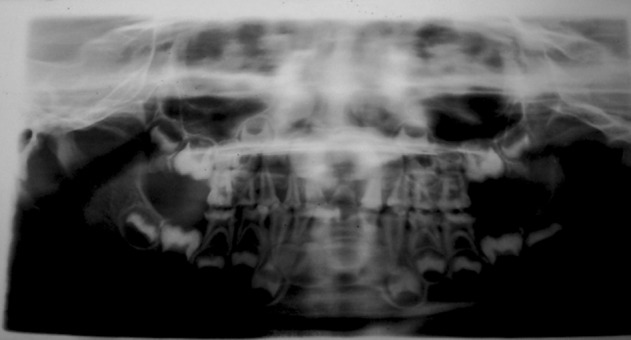
Orthopentamogram of the child (case 3) showing large pulp chambers and ‘shell like teeth’ and open canals in the upper anterior teeth

**Fig. 15.**
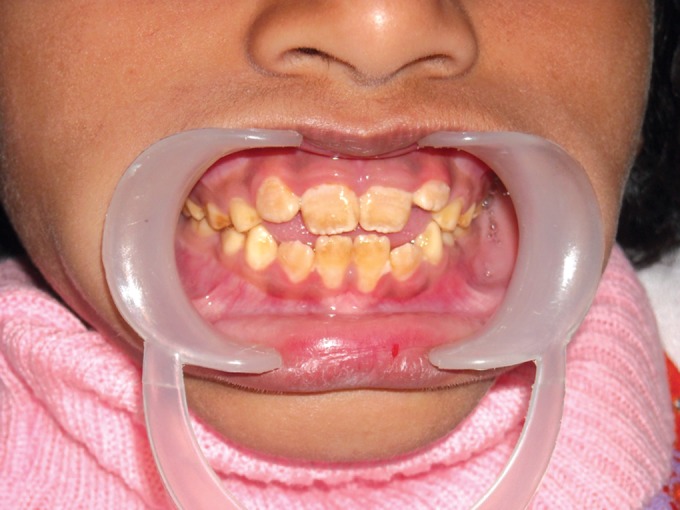
Photograph showing the yellowing and attrited dentition of 7 years old female (case 4)

**Fig. 16.**
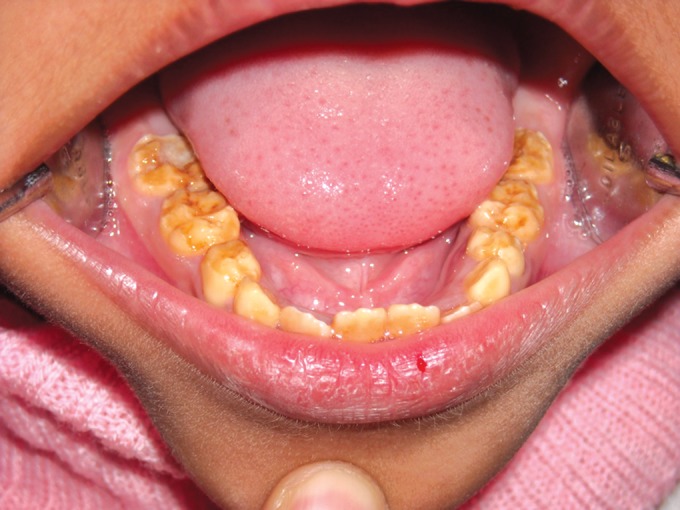
Photograph showing the yellowing and severely attrited permanent and deciduous mandibular teeth of the child (case 4)

**Fig. 17.**
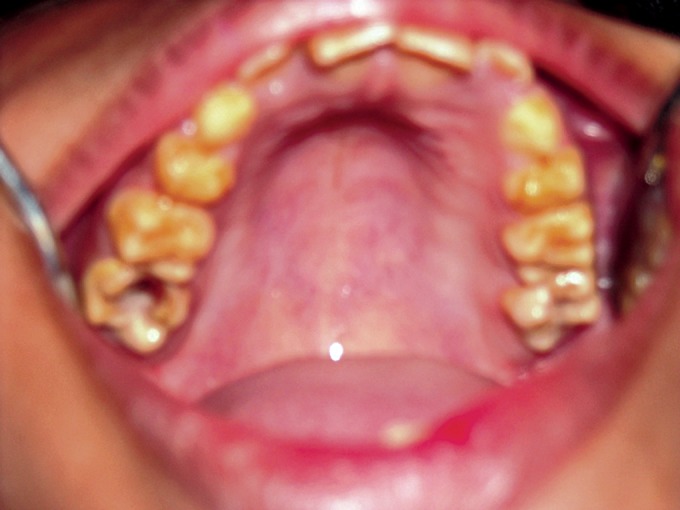
Photograph showing the yellowing and attrited permanent and deciduous maxillary teeth of the child (case 4); note the exposed 16, 26

**Fig. 18.**
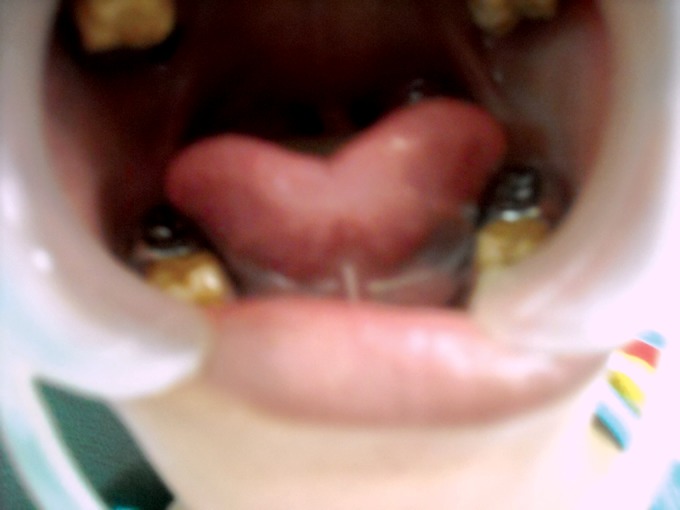
Photograph showing the management of multiple exposures of the molars with stainless steel crowns

Her father stated that teeth were all erupting discolored since she started teething and were wearing away gradually.

She was born of a consanguineous marriage but no history of such tooth abnormality was revealed, in other family members. Of her two younger siblings, one sister, aged 5 years had teeth of a normal morphology with no eruption anomalies; the other sister aged 7 months had just started teething.

Clinical examination revealed loss of enamel and dentin especially in the posterior teeth, both primary and permanent. The newly erupted and erupting permanent anteriors also showed the characteristic discoloration. The posterior teeth revealed hard dentin with worn occlusal surfaces, even up to the gingival third of the teeth, in permanent lower 6 of both sides. Maxillary left first molar showed early involvement with caries and was exposed and had been rendered nonvital.

### Radiographic Examination

Orthopantomograph revealed obliterated pulp chambers in most of teeth and constricted in some teeth. Then we went for full mouth IOPA radiographic survey which also showed the same findings.

## CASE 5

**Fig. 19.**
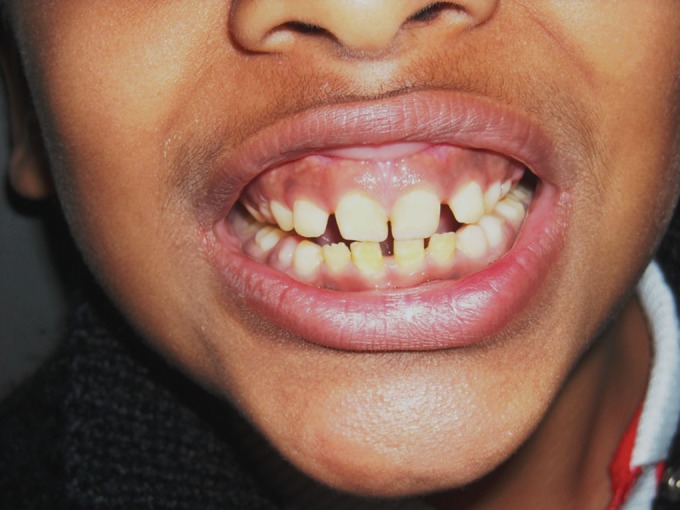
Photograph showing the mild yellowing and attrition of teeth of the child (case 5)

**Fig. 20.**
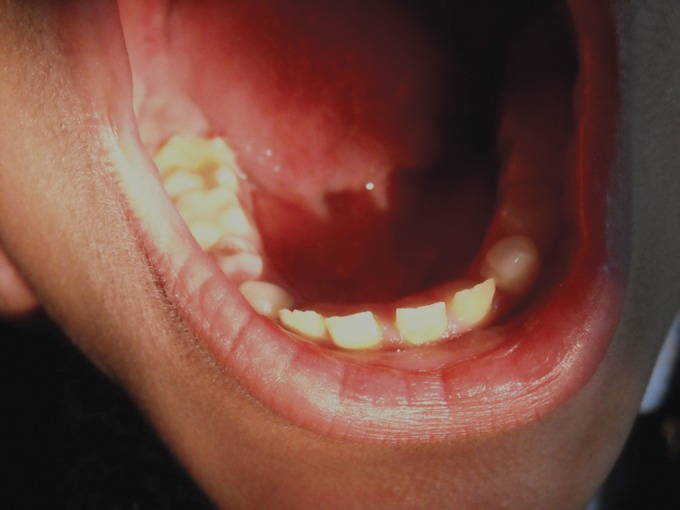
Photograph showing the mild yellowing attrition mandibular teeth of the child (case 5)

This patient, an 8 years old male, reported to the department, complaining of yellowing of teeth (Figs 19 and 20). He was accompanied by his father.

No history of such tooth abnormality was revealed, in any other family members.

Clinical examination revealed slight discoloration of the 4 permanent lower incisors and the permanent mandibular first molar teeth. There was some chipped enamel evident on the labial surface of the lower permanent incisors. The lower permanent molars were attrited occlusally. No other teeth were involved.

## CASE 6

**Fig. 21.**
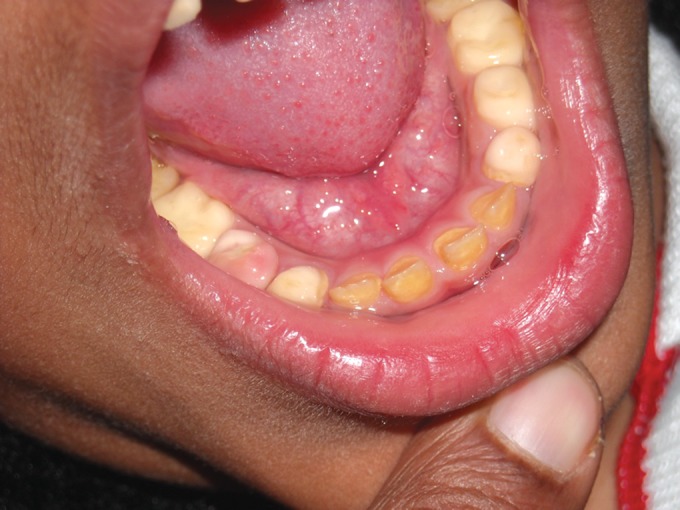
Photograph of the mandibular teeth of the 9 years old male (case 6) showing yellowed/attrided permanent incisors

In another case, a 9 years old male reported to our department, with the complaint of "lower front teeth coming out discolored" (Fig. 21).

He had been accompanied by his parents who gave a history of the discoloration of his teeth since the new permanent teeth erupted into oral cavity. His lower anterior milk teeth had been soft and easily worn off.

There was no family history of such tooth involvement.

Clinical examination revealed discolored yellow brown lower permanent anteriors (the central and lateral incisors) with soft enamel which readily gave way to probe pressure. Teeth revealed hard yellow brown dentin, incisal surfaces were sharp but labial and lingual surfaces revealed attrition already, up to the gingival level. Mandibular first deciduous molar of the right side showed the pinkish hue of internal resorbtion. The four permanent first molars and upper incisors were affected to a lesser degree.

## CASE 7

A boy aged 11 years came to our clinic, accompanied by his mother, seeking orthodontic treatment because of crowding of teeth and of gradually deviating lower jaw. The TMJ function was judged to be normal.

On examination it was found that he had class I malocclusion with crowding and multiple rotated teeth with some teeth erupting out of arch. Mild mottling and discoloration of teeth was observed. As per history the primary teeth had been normal.

The mother noted that her elder son (24 yrs) had had similar looking permanent teeth which gradually turned from dirty white to yellow and ultimately brown and started chipping off by age of 18 years. His lower anterior teeth had never erupted after the milk teeth fell off. The elder son had had extensive full mouth dental treatment for his ailment.

She herself had had similar looking teeth as a child and they too had turned in color from bad to worse and by age 20 had started chipping off. Her lower anteriors, like in her elder son’s case, had never erupted after the primary teeth fell off. Currently at age 42, most of her teeth had been attrited till the gingival third. She had a history of multiple extractions due to pulp exposure, and consequent swelling. She had had no restorative work done on the teeth. There was also a history of bone pain and treatment for calcium deficiency due to brittle bones. She indicated that lower right lateral incisor had erupted after the calcium therapy at age 28.

Out of her 5 siblings, her two sisters and one brother suffered similar dental symptoms. As did her father and her uncle and his son (but not his three daughters).

## DISCUSSION

Dentinogenesis imperfecta is one of the disorders that make the pediatric dentistry a challenging branch and pediatric dentist and important person for the patient. If a pediatric dentist is able to recognize this disorder of teeth that makes uncounted people into dental cripples, the loss of tooth substance can be minimized.

Recognizing dentinogenesis imperfecta in primary dentition not only helps the child to cope with the psychological trauma in childhood but also educating the patient and the parent regarding the treatment modalities for the permanent teeth, such as over dentures, stainless steel crowns, jacket crowns, pin retained cast gold "Thimbles" under acrylic resin crowns, stainless steel crowns with acrylic facing and simple removal appliances.^8^,^9^ Treatment during primary dentition is aimed more at preventive measures than definitive care. One should advice topical application of fluorides, pit and fissure sealants and maintainance of good oral hygiene to prevent the occurrence of caries. Stainless steel crowns can be used to prevent attrition of deciduous posterior teeth and young permanent teeth where aesthetics is not an issue. According to Wei such procedure must be undertaken as soon as the tooth erupts.^10^ Orthodontic treatment has been successfully performed in patients with different degrees of dentinogenesis imperfecta.^9^

Due to lack of hardness of dentin some authors specially Shafer et al emphasize that restorations cannot be permanent.^11^ Consequently when fractures occur at gingival level or below gum exodontia is indicated.^9^,^10^

## CONCLUSION

In these cases the we aimed at preserving the structure and function of teeth with stainless steel crowns (Fig. 18) for posterior teeth and aesthetic restoration of the anteriors, carious teeth were restored, root canal treatments were performed on the exposed teeth, drainage of abscess was done where indicated and antibiotics prescribed. Detailed counseling of the parents was done to ensure that regular dental care is provided to the child patient. And we emphasized and assured the parents that these children can also enjoy normal dental health like other children of their age.

## References

[1] Ten Cate (1998). R. Oral histology: development, structure and function.

[2] Barron MJ, McDonnell ST, MacKie I,  Dixon MJ (2008). Hereditary dentine disorders: Dentinogenesis imperfecta and dentine dysplasia. Orphanet J Rare Dis.

[3] Ten Cates (1998). R. Oral Histology, Development, structure and function.

[4]  Regezi JA,  Sciubba JJ,  Jordan RC Oral Pathology, clinical pathologic correlations.

[5] Kamboj M, Chandra  A (2007). Dentinogenesis imperfect type II: an affected family saga. J Oral Sci.

[6] Subramanium P, Mathew S, Sugnani SN (2008). Dentinogenesis
Imperfecta: A case report. J Indian Soc Pedod Prev Dent.

[7] Witkop CJ Jr. (1971). Manifestation of genetic diseases in human pulp. Oral Surg Oral Med Oral Pathol.

[8] Witkop CJ (1958). Genetics and dentistry. Eugen Quait.

[9] Mc Donald RE, Avery DR, Dean JA (2004). Dentisry for the child and adolescent.

[10] Wei SH (1988). Pediatric dentistry: Oral patient care.

[11] Shafer WG, Hine MK, Levy M, Tomich CE (1993). A textbook of Oral
pathology.

